# Diagnosis of peripheral pulmonary carcinoid tumor using endobronchial ultrasound

**DOI:** 10.4103/1817-1737.43082

**Published:** 2008

**Authors:** Daniel P. Steinfort, Moira Finlay, Louis B. Irving

**Affiliations:** *Department of Respiratory Medicine, Royal Melbourne Hospital, Victoria 3050, Australia*; 1 *Department of Pathology, Royal Melbourne Hospital, Victoria 3050, Australia*

**Keywords:** Pulmonary carcinoid tumor, endobronchial ultrasound, solitary pulmonary nodule

## Abstract

A 51-year-old woman with severe asthma underwent bronchoscopy and endobronchial ultrasound (EBUS) for investigation of a 15-mm peripheral lung nodule. Histology demonstrated a typical carcinoid tumor. Pulmonary location is the second commonest site for carcinoid tumors. Diagnosis of peripheral carcinoid tumor of the lung is difficult due to its small size, poor accuracy of cytologic diagnosis, and low sensitivity of positron emission tomography in detecting it. EBUS has a high diagnostic yield and a low complication rate in the evaluation of small solitary pulmonary nodules. The ultrasound appearance of carcinoid tumors is identical to that of lung carcinomas. Prompt diagnosis of carcinoid tumor is desirable as regional lymph node metastasis is seen in 10% of patients and is associated with a reduced 5-year survival. We feel that, where possible, all patients presenting with solitary pulmonary nodules should be investigated initially using EBUS due to its high diagnostic rate and the very low incidence of adverse events.

## Case Report

**Figure 1:**
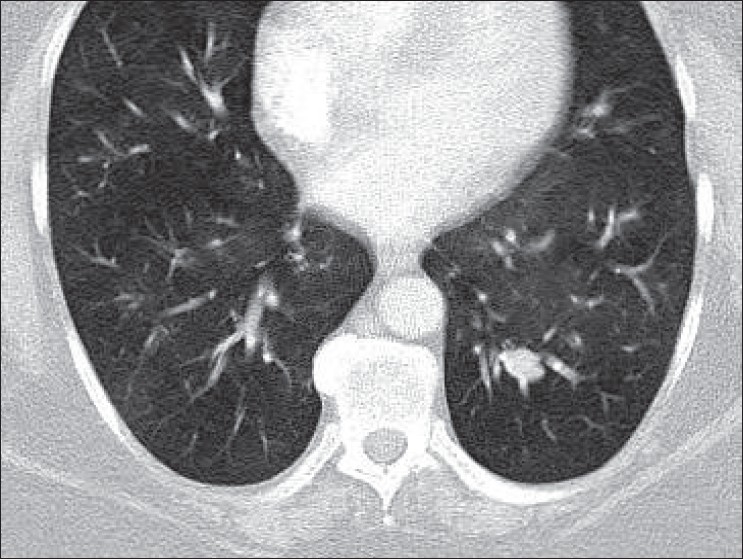
CT chest demonstrating left lower lobe nodule

A 51-year-old lady presented with persistently poor control of her asthma symptoms. She had a long-standing history of difficult asthma, frequently requiring oral prednisolone therapy. Respiratory function testing demonstrated moderate fixed airflow obstruction with a forced expiratory volume in 1 second (FEV_1_) of 1.0 liter. CT scanning of the chest was performed to exclude any factors that may have been responsible for exacerbating her illness. A lobulated, well-defined, soft tissue density nodule, measuring 15 mm was identified in the left lower lobe [Figure 1] but no other abnormality was noted.

**Figure 2:**
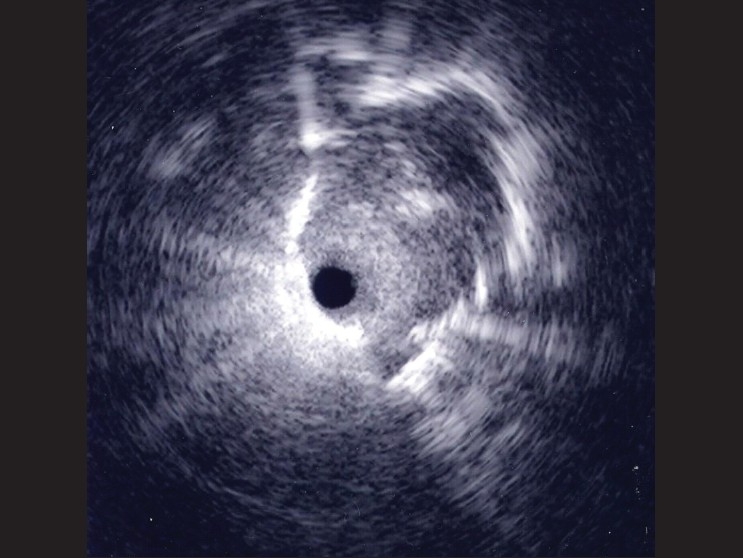
EBUS image obtained from left lower lobe

The patient underwent bronchoscopy with endobronchial examination via guide sheath (EBUS-GS). Examination in the posterobasal segment of the left lower lobe demonstrated a solid lesion [Figure 2]. Fluoroscopic examination of this region did not identify a mass. Transbronchial lung biopsy was performed via the guide sheath. The patient tolerated the procedure well and there were no complications.

**Figure 3:**
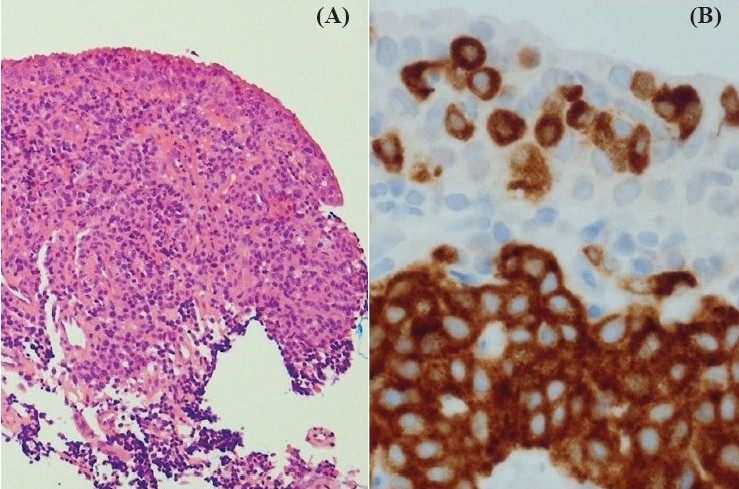
Histology from left lower lobe transbronchial biopsy; (A) hematoxylin and eosin ×10, (B) synaptophysin immunohistochemistry ×40

Histologic examination of biopsy specimens showed soft tissue infiltrated by sheets and cords of cells with slightly irregular nuclei, intranuclear inclusions, and fine granular chromatin[Figure 3A]. Immunohistochemistry demonstrated positive staining for synaptophysin [Figure 3B] and weakly positive staining for cytokeratin, chromogranin, synaptophysin, and TTF-1. Stains for S100 demonstrated sustentacular cells around clusters of neuroendocrine cells. A diagnosis of pulmonary carcinoid tumor was made.

## Discussion

Carcinoid tumors are low-grade malignancies comprising neuroendocrine cells. The commonest site for such lesions is the gastrointestinal tract, with a pulmonary location being the second commonest site.[1] They are rare tumors, with population-based studies indicating an incidence of 1 to 2/100,000 in different populations.[2,3]

The majority of pulmonary carcinoids are centrally located[4]; when located peripherally, biopsy is difficult as the lesion is frequently small. Our case highlights the ability of EBUS to achieve diagnosis in even very small peripheral lesions. No published literature describes the diagnostic yield of conventional bronchoscopy in peripheral carcinoid tumors. Diagnosis is made more difficult as the intact bronchial mucosa overlying the carcinoid tumor prevents cells from exfoliating. The diagnostic yield of cytology in carcinoid tumors is as low as 4[5] to 8%.[6]

EBUS has been demonstrated by numerous groups to improve the diagnostic evaluation of solitary pulmonary nodules (SPNs). Its value is most apparent with smaller lesions,[7,8] where the yield from transbronchial biopsy falls significantly. The diagnostic yield of conventional bronchoscopy in the investigation of lesions < 2 cm is as low as 14%.[9] The use of EBUS may enable correct diagnosis in up to 70% of cases with fluoroscopically invisible SPNs[10]; it has also been associated with a diagnostic yield of over 70% for lesions ≤ 20 mm[11] and of as much as 40% for lesions of ≤ 15 mm.[8]

Even large series examining the efficacy of EBUS report only very rare diagnosis of carcinoid tumour. Only one carcinoid was diagnosed in a cumulative 577 EBUS procedures.[10–13] Carcinoid tumors comprise 1–5% of all lung malignancies,[14,15] so these lesions are clearly underrepresented in EBUS cohorts. This significantly lower-than-expected proportion of carcinoids confirms the difficulties in bronchoscopic diagnosis of peripheral carcinoid tumors.

Without the histological diagnosis that was made possible by EBUS our patient may have gone undiagnosed for some more time. Firstly, over one-third of carcinoid tumors are diagnosed only at thoracotomy,[15] and this patient was not fit for such a procedure. Secondly, while the diagnostic accuracy of CT-guided needle biopsy is equivalent to EBUS,[16] pneumothorax rates are frequently greater than 40%[17,18] and increase with reducing lesion size.[19] In addition, cytologic diagnosis of carcinoid is problematic,[20] complicating use of fine needle aspiration for diagnosis. Thirdly, the sensitivity of PET scanning in carcinoid tumors is low, ranging around 75%,[21] which is thought to be a result of their small size and the hypometabolic state.

While 5-year survival from typical carcinoid tumors is as much as 91%,[14] delay in diagnosis is undesirable as regional lymph node involvement is seen in 10%[15] and is associated with adverse outcomes, even in typical carcinoid tumors.[14] Distant metastases are seen in 1.5% of cases.[15]

This case illustrates the significant value of bronchoscopy using EBUS in the investigation of small peripheral pulmonary nodules. Peripheral pulmonary carcinoid tumors may reliably be diagnosed using transbronchial biopsy, and their ultrasonographic appearance seems to be similar to other bronchogenic carcinomas. We feel that, where possible, all patients presenting with solitary pulmonary nodules should be investigated initially using EBUS due to its high diagnostic rate and very low incidence of adverse events.
